# Histological quantification of maize stem sections from FASGA-stained images

**DOI:** 10.1186/s13007-017-0225-z

**Published:** 2017-11-01

**Authors:** David Legland, Fadi El-Hage, Valérie Méchin, Matthieu Reymond

**Affiliations:** 1 UR1268 Biopolymères, Interactions et Assemblages, INRA, Nantes, France; 2 UMR 1318, Institut Jean-Pierre Bourgin, INRA-AgroParisTech, CNRS, Universite Paris-Saclay, Versailles, France

## Abstract

**Background:**

Crop species are of increasing interest both for cattle feeding and for bioethanol production. The degradability of the plant material largely depends on the lignification of the tissues, but it also depends on histological features such as the cellular morphology or the relative amount of each tissue fraction. There is therefore a need for high-throughput phenotyping systems that quantify the histology of plant sections.

**Results:**

We developed custom image processing and an analysis procedure for quantifying the histology of maize stem sections coloured with FASGA staining and digitalised with whole microscopy slide scanners. The procedure results in an automated segmentation of the input images into distinct tissue regions. The size and the fraction area of each tissue region can be quantified, as well as the average coloration within each region. The measured features can discriminate contrasted genotypes and identify changes in histology induced by environmental factors such as water deficit.

**Conclusions:**

The simplicity and the availability of the software will facilitate the elucidation of the relationships between the chemical composition of the tissues and changes in plant histology. The tool is expected to be useful for the study of large genetic populations, and to better understand the impact of environmental factors on plant histology.

**Electronic supplementary material:**

The online version of this article (doi:10.1186/s13007-017-0225-z) contains supplementary material, which is available to authorized users.

## Background

Crop species like maize (*Zea mays* L.) are of increasing interest both for cattle feeding [1] and for bioethanol production [2–5]. The polysaccharidic fraction, mainly composed of the stem and the leaf cell walls, is digested or transformed into energy or fuel after several mechanical, biochemical and/or enzymatic processes. Many studies have been devoted to the elucidation of relationships between cell wall chemical composition and degradability [3, 6–9]. The lignin content is a key factor for explaining degradability. Several authors have reported that the variations of lignification according to the tissue may explain differences in the digestibility of plants at similar maturity stages [4, 10–12]. There is therefore a need to better understand variations in tissue lignification within stems and their relationships with external factors such as water availability or genotype.

Differences in the biochemical composition of tissues may be assessed by several methods. Manual dissection of tissues makes it possible to compare their biochemical composition and degradability [12]. However, these micro dissections entail very tedious work [3, 12] and are limited to an a priori choice of specific tissues.

The continuous development of imaging techniques has led to promising ways for investigating the chemical composition of plant tissues. Fluorescence imaging techniques allow for the localisation of specific proteins, polysaccharides and phenolic compounds [13, 14]. Vibrational microspectroscopies such as infra-red or Raman spectroscopies provide complementary information about the molecular composition of the observed materials [15]. However, the imaging of plant tissues is usually performed within a small field of view, making it difficult to quantify the variations of compositions within an organ such as a stem. X-ray computed (micro-) tomography is a powerful tool that enables the acquisition of 3D images of a whole sample, and several studies have been performed on plant materials [16–18]. However, the lack of information about chemical composition of cell walls limits the differentiation of lignified and non-lignified tissues. Mass spectrometry imaging has shown promising results for the visualisation of the distribution of specific chemical structures within a whole slice [19, 20]. The spatial resolution is larger than that of microscopy.

Plant cross-section staining is an alternative that can reveal components on a thin stem cross-section [9, 21–27]. In a previous study, Méchin et al. [5] compared the use of Maüle [28], phloroglucinol [29] and FASGA stainings [30] to assess the global lignification of maize stem cross-sections. FASGA staining coupled with image analysis was shown to be the best-adapted method. FASGA stains lignified tissues in red, whereas non-lignified or poorly lignified tissues are stained in blue. In a recent study, Zhang et al. [31] proposed an automated image analysis method for quantifying the histology of FASGA-stained sections of maize stems. However, the resolution of the images did not allow an accurate estimation of the proportion of the various tissue types, and it was not possible to quantify the histology of the different tissues.

In order to increase the resolution of acquired images, microscopy slide scanners provide promising features. They allow the scanning of an entire whole mounted histology sample, with a resolution of a few microns [32], making it possible to observe cellular morphology and organisation at the scale of the whole organ, with a resolution comparable to that of microscopy. It consequently appears to be a method of choice for the quantitative analysis of lignification within a whole stem section. The recording of the colorimetric information provided by the staining makes it possible to develop an automated image analysis procedure [33]. However, the huge amount of data generated increases the difficulty of processing and analysis [34].

The aim of this work is to present an automated method for the analysis of stained images of stem sections observed with a microscopy slide scanner. The method combines the identification of the different tissue regions that constitute the section based on colorimetric and morphological information, the quantification of the morphometry and the colorimetry of each tissue region. The method is illustrated on a collection of stem sections from several genotypes obtained with contrasted growing conditions.

## Methods

### Sample preparation

Four maize inbred lines (Cm484, F4, F271 and F7025) were selected from preliminary experiments performed at INRA Lusignan between 2006 and 2008 [2]. Plants were cultivated in Mauguio (southern France) during the years 2013 and 2014. Two different irrigation scenarios were used: one with irrigation, and the other one without irrigation where the watering was stopped after appearance of the 5th “liguled” leaf on the plant of a reference genotype and then restarted 14 days after flowering (i.e., from June to August without artificial watering). For each condition (with or without irrigation) the trials were randomised block designs with two replicates. The length of the rows was 4.20 m, the inter-row spacing was 0.80 cm and the density was 80,000 plants per hectare. The whole internode located under the main ear was collected for three plants at the silage stage (~ 30% of dry matter content) for each condition, block and genotype. Internodes were stored in 70% ethanol before quantitative histological analysis.

### Image acquisition and preparation

A 1-cm-long segment was sampled in the upper part of each internode. For each segment, 15 cross-sections with a thickness of 150 µm were prepared using an HM 650 V Vibratome from MicroMicrotech France. Sections were stained for 24 h using a FASGA solution diluted in distilled water (1:8, v/v). The FASGA solution was composed of 0.05% safranin O, 0.2% Alcian blue, 1.5% acetic acid and 46% glycerine in distilled water. Safranin is a red, basic, cationic dye, and Alcian blue is an acidic anionic dye. Because lignin is acidic (due to its phenolic hydroxyl groups), lignified tissues are stained in red even if this stain is not completely specific for only lignin. FASGA thus stains lignified tissues in red, whereas non-lignified or poorly lignified tissues appear as blue. After staining, sections were rinsed for 24 h with distilled water while stirring continuously.

**Fig. 1:**
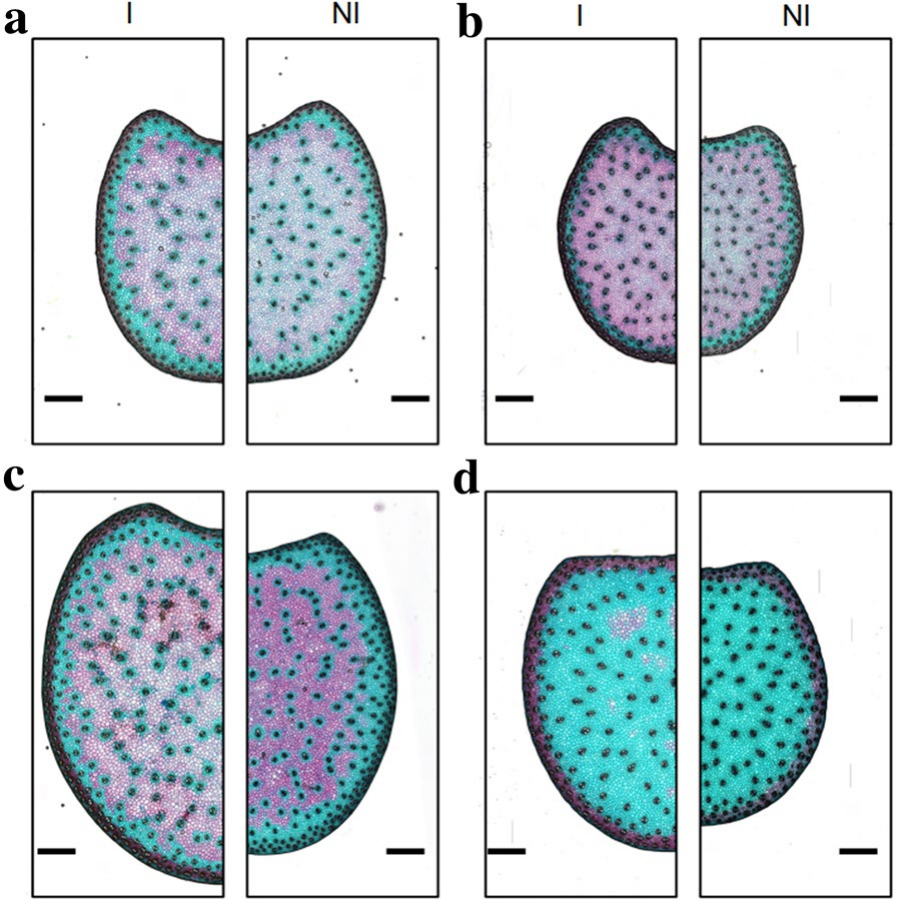
Sample images of FASGA-stained cross sections of maize stems showing the heterogeneity of coloration for four genotypes and two irrigation conditions. **a**: Cm484; **b**: F271; **c**: F7025; **d**: F4. Left-hand panels correspond to the irrigated condition, right-hand panels to the non-irrigated condition

An image of each cross-section was acquired using a slide scanner piloted by the Metafer scanning and imaging platform (MetaSystems GmbH, Altlussheim, Germany). The complete system is composed of an AxioImager Z2 Zeiss microscope equipped with a CoolCube 1 camera, a robotic system consisting of a rotating feeder module that delivers samples to the microscope stage, and a computer piloted by Metafer software. Each image was acquired with the 5× objective lens. To reduce computation time and memory space, each picture was converted into a plain TIFF image by choosing the 6× zoom with MetaViewer software. Resulting images had a size of 4000 × 4000 pixels approximately and a resolution of 5.17 µm per pixel. Sample images for each genotype and each water treatment are presented in Fig. 1.

### Image processing

**Fig. 2:**
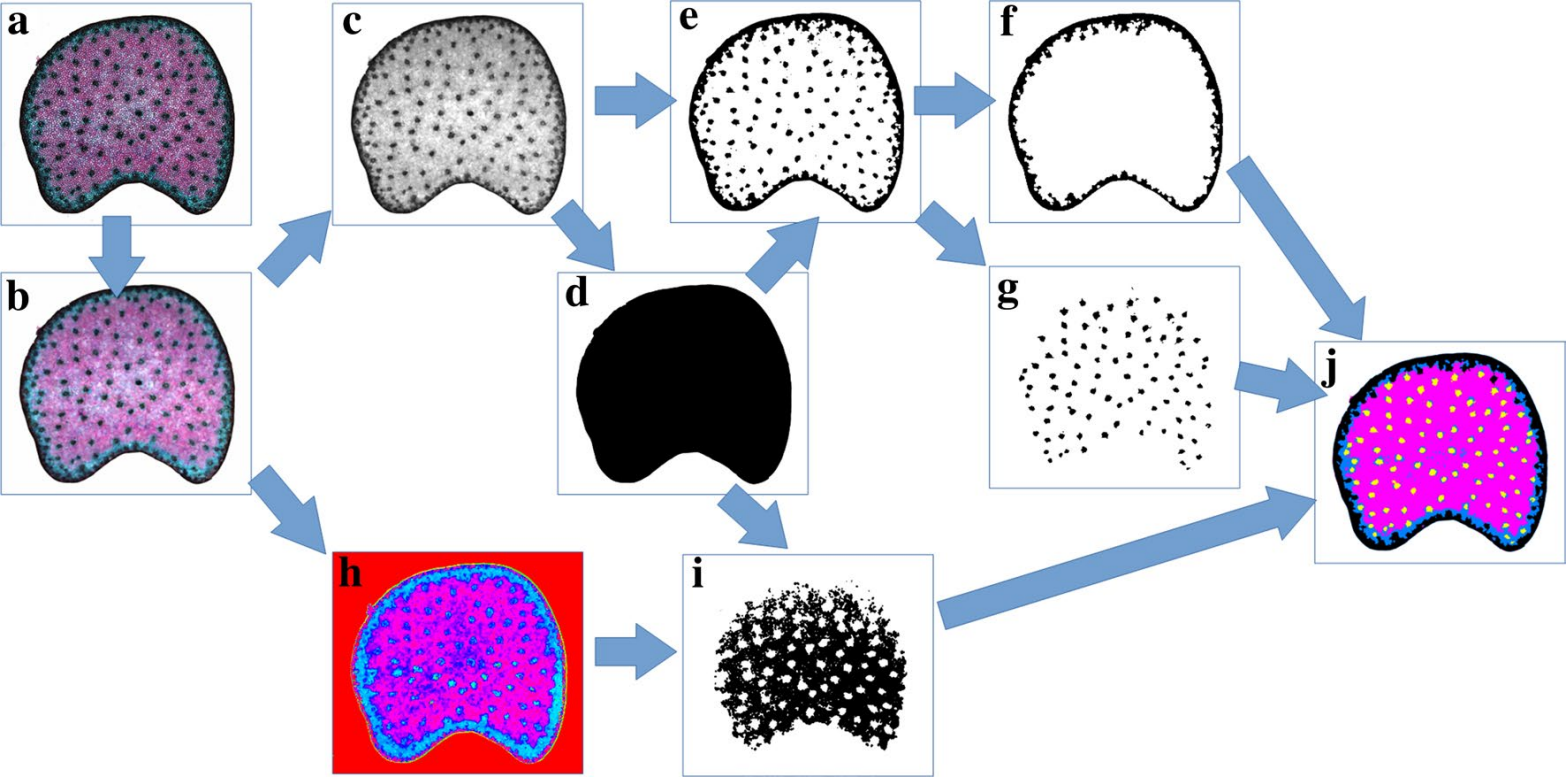
Image segmentation workflow. **a** Original image. **b** Result of morphological filtering and Gaussian smoothing. **c** Luminance image. **d** Stem mask computation. **e** Dark structures identification. **f** Rind identification. **g** Bundles identification. **h** Colour representation of the hue image. **i** Identification of the lignified regions. **j** Result of composite image

A fully automated image processing workflow was designed for identifying the different tissue regions that compose the internode sections. The workflow is summarised in Fig. 2.

Filtering was first applied to the image to enhance the colour contrast and reduce acquisition noise. A combination of morphological opening and closing was applied [35], followed by a Gaussian smoothing (Fig. 2b).

The processing of colour images often takes advantage of transforming the RGB colours into a colour space that better discriminates the colours, such as HSV or Lab colour spaces [36–38]. The segmentation of the different tissue regions was based on the hue and luminance images. The hue represents the pure colour of a pixel, and the luminance quantifies the brightness.

The luminance image was used to identify the stem section (Fig. 2c). A hysteresis thresholding was applied to identify the regions of the stem occupied by tissues, while removing the regions corresponding to holes. Air bubbles could be observed on some images, resulting in thin dark artefacts outside of the stem. An additional morphological closing was added to remove eventual bubble boundaries. The result was a binary mask, used for restricting further processing of the valid regions (Fig. 2d).

The highly lignified tissues corresponding to vascular bundles and to the rind were identified by thresholding low values in the luminance image (Fig. 2e). Area opening was applied to remove segmentation noise [35]. A connected component labelling was used to identify the largest region that corresponded to the rind (Fig. 2f). Since some bundles could be connected to the region corresponding to the rind, a morphological opening was applied to separate them from the rind. From the luminance image it was then possible to identify the rind (containing outer vascular bundles) and the vascular bundles of the pith (Fig. 2g).

The hue component of the filtered colour image was used to discriminate between highly and lowly lignified regions of the pith parenchyma (Fig. 2h). The application of a threshold image could discriminate between lignified tissues (red-magenta colour) and non-lignified tissues (light cyan colour) (Fig. 2i).

Finally, the elementary binary images corresponding to the different tissue regions (rind, pith vascular bundles, lignified pith parenchyma, non-lignified pith parenchyma) were combined to create a label image used for histology quantification. A colour image showing each tissue with a specific colour was eventually used for final validation (Fig. 2j).

### Image analysis

Several morphometric descriptors were computed from the label images corresponding to segmented tissue regions. The total area and the area fraction of each tissue region were computed on each image. The average values of the red, green and blue channels were also computed for each tissue region. The number of pith vascular bundles was counted automatically on each section. The ratio of the number of vascular bundles over the area of the section led to a measurement of vascular bundle intensity. In total, 19 descriptors were obtained (Table 1). Seven of them corresponded to morphometric features (area, area fractions of tissue regions, or bundle number). The remaining 12 descriptors corresponded to the measure of colorimetry in a specific tissue region.

**Table 1:** List and description of the 19 descriptors obtained by automated image analysis and used for statistical analysis

Feature name	Description	Type
Stem area	The area occupied by the stem section, in cm^2^	Morphometry
Bundle number	The number of vascular bundles in the pith	
Bundle intensity	The numerical intensity of bundles, in cm^−2^	
Lignified fraction	The tissue fraction corresponding to lignified pith	
Non lignified fraction	The tissue fraction corresponding to non-lignified pith	
Rind fraction	The tissue fraction corresponding to the rind	
Bundle fraction	The tissue fraction corresponding to vascular bundles in pith	
Lignified mean red	The mean red intensity in the lignified fraction	Colorimetry
Lignified mean green	The mean green intensity in the lignified fraction	
Lignified mean blue	The mean blue intensity in the lignified fraction	
Non-lignified mean red	The mean red intensity in the non-lignified fraction	
Non-lignified mean green	The mean green intensity in the non-lignified fraction	
Non-lignified mean blue	The mean blue intensity in the non-lignified fraction	
Rind mean red	The mean red intensity in the rind	
Rind mean green	The mean green intensity in the rind	
Rind mean blue	The mean blue intensity in the rind	
Bundles mean red	The mean red intensity in the vascular bundles	
Bundles mean green	The mean green intensity in the vascular bundles	
Bundles mean blue	The mean blue intensity in the vascular bundles	

### Software implementation

The whole image processing workflow was developed within the ImageJ/Fiji plateform [39], using the MorphoLibJ library [40]. The whole workflow was implemented as an ImageJ/Fiji plugin, freely available on the Internet [41]. The plugin provides the possibility to finely tune the different parameters used at each step of the workflow. A macro is also provided, making it possible to process a whole batch of images using the same parameters.

### Statistical analyses

Statistical analyses were performed within the Matlab software (The Mathworks, Natick, MA, USA), using the statistics toolbox and the MatStats library, a collection of functions developed in-house to facilitate the exploration and the analysis of statistical data sets [42].

Analyses of variance were performed by applying a general linear model to each of the 19 descriptors. Each model took the fixed effects of the genotype, the water treatment and the year, their interactions, the random effect of the sampling block nested to fixed effects, and the random effect of the stem nested to the block into account. The model for a descriptor *f* is given by the following equation:

$${\text{f}}_{\text{ijklm}} = {\upmu } + {\text{G}}_{\text{i}} + {\text{T}}_{\text{j}} + {\text{Y}}_{\text{k}} + \left( {\text{GT}} \right)_{\text{ij}} + \left( {\text{GY}} \right)_{\text{ik}} + \left( {\text{TY}} \right)_{\text{jk}} + \left( {\text{GTY}} \right)_{\text{ijk}} + {\text{B}}_{\text{l}} \left( {{\text{GTY}}_{\text{ijk}} } \right) + {\text{S}}_{\text{m}} \left( {{\text{GTYB}}_{\text{ijkl}} } \right) + \upvarepsilon_{\text{ijklm}}$$

where µ is the constant, G_i_, T_j_ and Y_k_ are the fixed effects of the genotype, the water treatment and the year, respectively, (GT)_ij_, (GY)_ik_, (TY)_jk_ and (GTY)_ijk_ are the fixed effects of the interactions up to the third order, B_l_(GTK_ijk_) is the random effect of the block nested to the genotype, the treatment and the year, S_m_(GTYB_ijkl_) is the random effect of the stem nested to all other effects, and ε_ijklm_ is the residual error term. The “anovan” function of the statistics toolbox of Matlab was used for each of the 19 models. The resulting *p* values of all the models were concatenated in a data table using the descriptors as rows and the effects as columns.

## Results and discussion

### Acquisition of digital images

Figure 1 shows images of sample sections from each genotype and each irrigation condition. The size of sections depends largely on the genotype. After FASGA staining, lignified tissues appear in red, whereas non-lignified tissues appear in light blue. The vascular bundles and the rind usually appear in dark red or brown. Except for one genotype that generally appears to be non-lignified, a peripheral blue ring located below the rind was observed. On some sections, a blue ring around vascular bundles corresponding to non-lignified tissues can be clearly recognised.

The genotypes present clearly distinct responses to the coloration. For instance, the F4 genotype shows a small amount of lignified parenchyma. Variations in section size and in proportion of the tissue regions may also be observed. For each genotype, some variations in histology may be distinguished between irrigated and non-irrigated conditions. For example, section size seems to be smaller as does rind thickness in the case of water deficit. However, a large variability in the size and shape of the sections may be observed.

### Segmentation of FASGA images

**Fig. 3:**
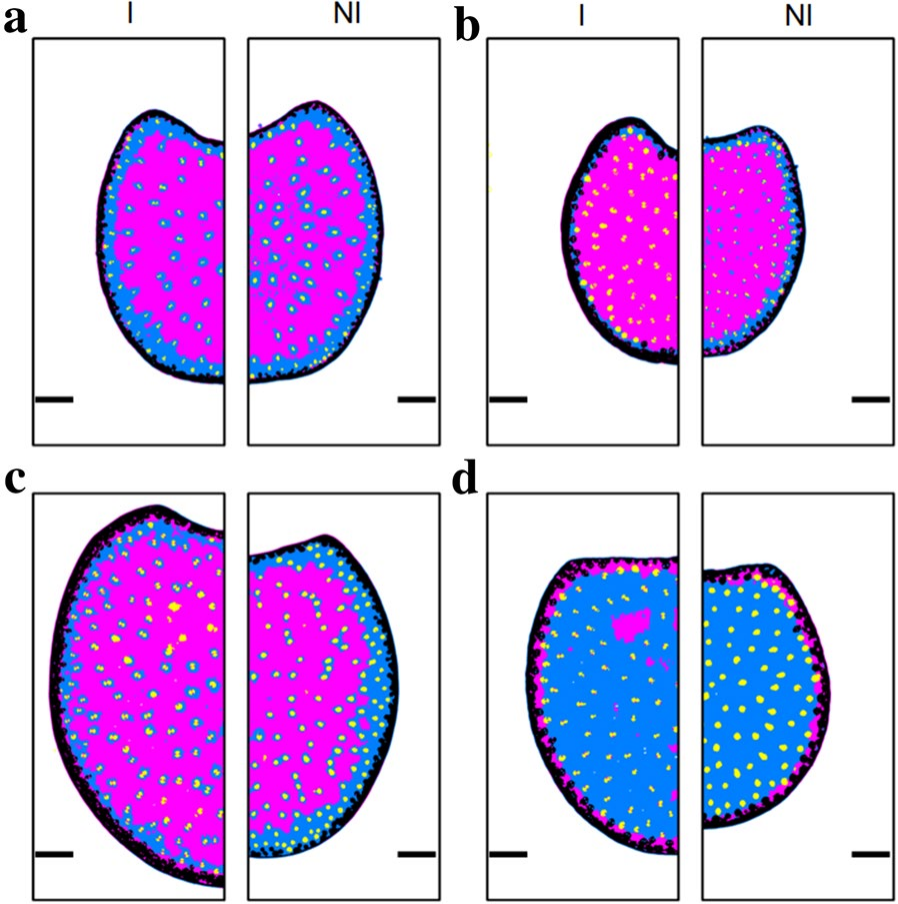
Result of automated labelling of tissue regions from FASGA-stained sections. Each tissue region is represented with a different colour. Black: rind; yellow: vascular bundles; magenta: lignified tissues; blue: non-lignified tissues. **a**: Cm484; **b**: F271; **c**: F7025; **d**: F4. Left-hand panels correspond to the irrigated condition, right-hand panels to the non-irrigated condition

Figure 3 shows sample results of the automated labelling of tissues from FASGA stained sections. The segmented images correspond to the images presented in Fig. 1. The lignified and the non-lignified tissues can be clearly discriminated. The peripheral rind can also be identified, as well as the inner ring of lowly lignified parenchyma. The variations of rind thickness can be better distinguished after automated labelling of tissues. The vascular bundles are nearly all identified. The vascular bundles located within or close to the rind are difficult to separate from the rind.

### Quantification of histology

The main results of the linear models applied to each descriptor are presented in Table 2.

**Table 2:** Results of the analysis of variance performed on each descriptor

Name	G	T	Y	B (G × T × Y)	S (G × T × Y × B)	G × T	G × Y	T × Y	G × T × Y
Morphometry									
Stem area (cm^2^)	0.000***	0.005**	0.196	0.958	0.000***	0.464	0.002**	0.189	0.156
Bundle number	0.000***	0.578	0.219	0.013*	0.000***	0.661	0.188	0.606	0.565
Bundle intensity	0.000***	0.001**	0.472	0.748	0.000***	0.432	0.043*	0.172	0.268
Tissue fractions									
Lignified fraction	0.000***	0.024*	0.826	0.090	0.000***	0.036*	0.406	0.901	0.592
Non lignified fraction	0.000***	0.079	0.154	0.773	0.000***	0.062	0.386	0.838	0.019*
Rind fraction	0.007**	0.542	0.290	0.011*	0.000***	0.394	0.022*	0.198	0.318
Bundle fraction	0.000***	0.009**	0.400	0.050	0.000***	0.402	0.221	0.073	0.904
Lignified fraction									
Mean red	0.000***	0.003**	0.008**	0.143	0.000***	0.195	0.009**	0.484	0.281
Mean green	0.000***	0.328	0.411	0.473	0.000***	0.462	0.058	0.088	0.032*
Mean blue	0.000***	0.125	0.841	0.567	0.000***	0.201	0.037	0.087	0.031*
Non-lignified fraction									
Mean red	0.004**	0.040*	0.134	0.234	0.000***	0.013*	0.027*	0.395	0.685
Mean green	0.000***	0.160	0.002**	0.535	0.000***	0.836	0.061	0.175	0.005**
Mean blue	0.000***	0.009**	0.034	0.334	0.000***	0.530	0.039*	0.241	0.006**
Rind fraction									
Mean red	0.021*	0.001**	0.040*	0.174	0.000***	0.228	0.006**	0.727	0.031*
Mean green	0.000***	0.004**	0.001**	0.331	0.000***	0.024*	0.002**	0.008**	0.272
Mean blue	0.001**	0.256	0.006**	0.167	0.000***	0.099	0.002**	0.032*	0.289
Bundle fraction									
Mean red	0.002**	0.000***	0.250	0.032*	0.132	0.213	0.004**	0.685	0.104
Mean green	0.023*	0.001**	0.032*	0.021*	0.171	0.492	0.791	0.722	0.288
Mean blue	0.716	0.000***	0.146	0.027*	0.492	0.082	0.059	0.335	0.342

The table provides the *p* values for each fixed effect of the main factors (genotype, water treatment, year), their interactions, and for the random effects of the block and of the stem. *p* values lower than 0.05, 0.01 and 0.001 are indicated with *, ** and ***, respectively. *p* values lower than 0.01 are in italic type

For most features, the genotype effect is highly significant (*p* values lower than 0.01 for 12 features). This validates the methodology for discriminating contrasted genotypes based on quantitative histology.

The effect of the water treatment is significant for several descriptors as well. In particular, the area of the section and the area fraction occupied by the bundles vary with the water treatment. The water treatment also has a significant effect on the coloration of the tissue fraction. The interaction of the genotype and water treatment effect is significant on the fraction of lignified tissue (*p* value ≈ 0.036), on the mean red value of lignified regions (*p* value ≈ 0.013), and on the mean green value of the rind tissue (*p* value ≈ 0.024). This can be interpreted as differentiated responses of each genotype to the water treatment.

The average value for each morphometric descriptor is given in Table 3. In addition to the global average value, the average value by combination of genotype and water treatment is also provided.

**Table 3:** Average value of each parameter measured on cross-section images

	Global	Genotypes							
		Cm484		F271		F4		F7025	
		I	NI	I	NI	I	NI	I	NI
Count	125	16	16	14	15	16	16	16	16
Morphometry									
Stem area (cm^2^)	1.81 *(0.75)*	1.75 *(0.22)*	1.64 *(0.21)*	1.29 *(0.18)*	1.02 *(0.19)*	1.55 *(0.41)*	1.44 *(0.21)*	3.02 *(0.79)*	2.66 *(0.42)*
Bundle number	153.2 *(46.2)*	114.7 *(9.4)*	128.8 *(20.0)*	143.1 *(27.9)*	134.6 *(33.0)*	129 *(23.9)*	147.9 *(32.3)*	214.1 *(38.9)*	211.3 *(38.7)*
Bundle intensity	91.2 *(26.9)*	66.6 *(10.1)*	79.8 *(14.2)*	111.2 *(17.2)*	133.8 *(28.6)*	85.3 *(10.7)*	103.3 *(17.1)*	74.5 *(18.7)*	80.5 *(17.5)*
Tissue fractions									
Lignified fraction	51.9 *(24.5)*	61.1 *(2.7)*	65.3 *(3.5)*	72.8 *(4.0)*	68.9 *(2.4)*	15.6 *(9.1)*	8.2 *(2.3)*	68.1 *(4.5)*	58.6 *(9.1)*
Non lignified fraction	30.1 *(25.3)*	23.8 *(3.7)*	20.9 *(4.0)*	6.8 *(3.3)*	8.9 *(3.8)*	68.3 *(9.6)*	73.4 *(5.9)*	14.2 *(3.8)*	19.8 *(7.2)*
Rind fraction	12.9 *(3.9)*	12 *(0.9)*	10.3 *(1.4)*	15.9 *(3.1)*	16.8 *(4.5)*	11.6 *(3.0)*	11.5 *(4.1)*	11.7 *(2.2)*	14.2 *(5.3)*
Bundle fraction	5.2 *(2.0)*	3.1 *(0.8)*	3.5 *(0.7)*	4.5 *(1.0)*	5.5 *(1.4)*	4.5 *(1.1)*	6.9 *(2.0)*	6.1 *(1.6)*	7.3 *(2.2)*
Lignified fraction									
Mean red	157.8 *(29.0)*	182.1 *(7.3)*	182.6 *(6.7)*	173.3 *(5.9)*	159.3 *(4.1)*	124.3 *(25.8)*	107.3 *(19.2)*	172.5 *(6.7)*	162.8 *(5.3)*
Mean green	145.6 *(25.6)*	174.2 *(13.5)*	177.7 *(8.8)*	147.1 *(10.0)*	146.3 *(9.2)*	127.4 *(27.8)*	114.7 *(23.5)*	140 *(13.3)*	137.6 *(8.1)*
Mean blue	180 *(24.7)*	204.9 *(8.6)*	207.1 *(4.2)*	184.8 *(6.7)*	178.2 *(6.5)*	158.1 *(27.4)*	143 *(23.0)*	181.6 *(9.0)*	182.4 *(6.2)*
Non-lignified fraction									
Mean red	70 *(13.3)*	76.7 *(9.0)*	78.7 *(9.0)*	66 *(11.4)*	74.3 *(9.1)*	78.6 *(11.7)*	61.3 *(12.4)*	68.9 *(12.7)*	55.2 *(9.8)*
Mean green	149.8 *(31.3)*	155.5 *(15.3)*	153.3 *(11.6)*	113.2 *(14.9)*	115.4 *(17.6)*	195 *(7.7)*	186.5 *(19.6)*	138.3 *(10.3)*	134.5 *(14.4)*
Mean blue	156.6 *(28.6)*	157.8 *(12.8)*	156.4 *(8.8)*	126.9 *(13.1)*	119.1 *(20.7)*	200.1 *(4.8)*	188.6 *(15.6)*	153.3 *(7.2)*	145 *(12.0)*
Rind fraction									
Mean red	40.2 *(7.6)*	43.3 *(6.4)*	42 *(6.0)*	45.2 *(5.3)*	39.1 *(12.0)*	43.7 *(4.4)*	37 *(6.6)*	41 *(3.1)*	30.9 *(4.4)*
Mean green	42.9 *(7.0)*	43.8 *(3.3)*	48.3 *(5.6)*	42 *(3.3)*	43.5 *(9.3)*	45.8 *(6.5)*	45.5 *(5.0)*	32.5 *(3.5)*	41.8 *(4.6)*
Mean blue	50.4 *(6.7)*	47.7 *(2.6)*	52.1 *(4.9)*	50.3 *(4.3)*	48.6 *(11.0)*	56.2 *(6.5)*	54.4 *(4.8)*	44.1 *(4.1)*	49.3 *(4.5)*
Bundle fraction									
Mean red	43.4 *(7.4)*	42.7 *(3.7)*	39.4 *(3.8)*	53.2 *(4.3)*	44.3 *(5.9)*	45.4 *(3.7)*	36.9 *(6.3)*	48.4 *(8.4)*	38 *(5.8)*
Mean green	59.2 *(6.8)*	65.4 *(4.5)*	60.5 *(4.6)*	62.2 *(5.3)*	52.9 *(7.7)*	62.2 *(6.4)*	57.3 *(4.4)*	58.1 *(6.1)*	54.9 *(6.0)*
Mean blue	65.4 *(7.0)*	67.3 *(3.5)*	62.7 *(4.6)*	73.7 *(6.2)*	59.5 *(9.3)*	66.7 *(5.7)*	61.7 *(5.3)*	68.7 *(4.9)*	63.3 *(5.4)*

For each parameter, the table presents the global average as well as the average for each combination of genotype and water treatment. The standard deviations are given in brackets

### Comparison of genotypes

Several histological parameters are useful for discriminating the genotypes. In the following section, the genotypes are compared based on the irrigated condition. The area of the section is approximately 1.81 cm^2^ (SD 0.75), with a significant effect of the genotype (*p* value < 1e−3). It is larger for the F7025 line (3.02 cm^2^, SD 0.79) and smaller for the F271 genotype (1.29 cm^2^, SD 0.18).

F7025 has the largest number of vascular bundles (214.1, SD 38.9), whereas the average value is 153.2 (SD 46.2). However, F7025 is also the genotype with the largest sections. When considering the numerical density of vascular bundles per unit area, the F7025 genotype is comparable to the genotypes Cm484 and F4 (74.5, 66.6 and 85.3 cm^−2^, respectively, with SD ranging from 10.1 to 18.7). In that case, the increase in the number of bundles seems to be a direct consequence of the increase in the size of the section. On the contrary, the genotype F271 presents larger bundle intensity (111.2 cm^−2^, SD 17.2 cm^−2^).

The fraction of lignified tissue is around 60–75% for most genotypes except for the F4 genotype whose lignified fraction is 15.6% (SD 9.1%). This corresponds to the large proportion of blue area that can be observed on the segmented images for this genotype (Fig. 3). F271 presents the largest fraction of lignified tissue (72.8%, SD 4.0%). It is also the one with the largest rind fraction (15.9%, SD 3.1%), whereas the average rind fraction is around 12.9% (SD 3.9%) among the studied genotypes in both conditions. The lignified fraction of the F4 genotype appears to be darker than the other genotypes. The mean red value for F4 is 124.3 (SD 25.8), compared to that of the global average of 157.8 (SD 29.0). Similar variations occur for the green and blue mean values. On the contrary, the non-lignified fraction of the F4 genotype appears to be lighter. The mean green or blue values are 195 (SD 7.7) and 200.1 (SD 4.8), whereas they are 149.8 (SD 31.3) and 156.6 (SD 28.6) on average for the studied genotypes in both irrigation conditions. These two observations may indicate a differentiation in the lignification of the tissues for the F4 genotype.

Depending on the genotypes, some tissues may present specific colour variations. The rind of F7025 is slightly darker than the rind of the other genotypes. For instance the mean green value is 32.5 (SD 3.5) for F7025, whereas the average values for other genotypes are around 42–45.8 (global SD 7.0). This can be interpreted as an increase in the lignification of the rind of F7025. The vascular bundles of the F271 genotype appear to have a greater value in the red component. The mean red value is 53.2 (SD 4.3), and ranges between 42.7 and 48.4 (global SD 7.4) for other genotypes. This suggests an increase of lignification in the bundles of the F271 genotype.

**Fig. 4:**
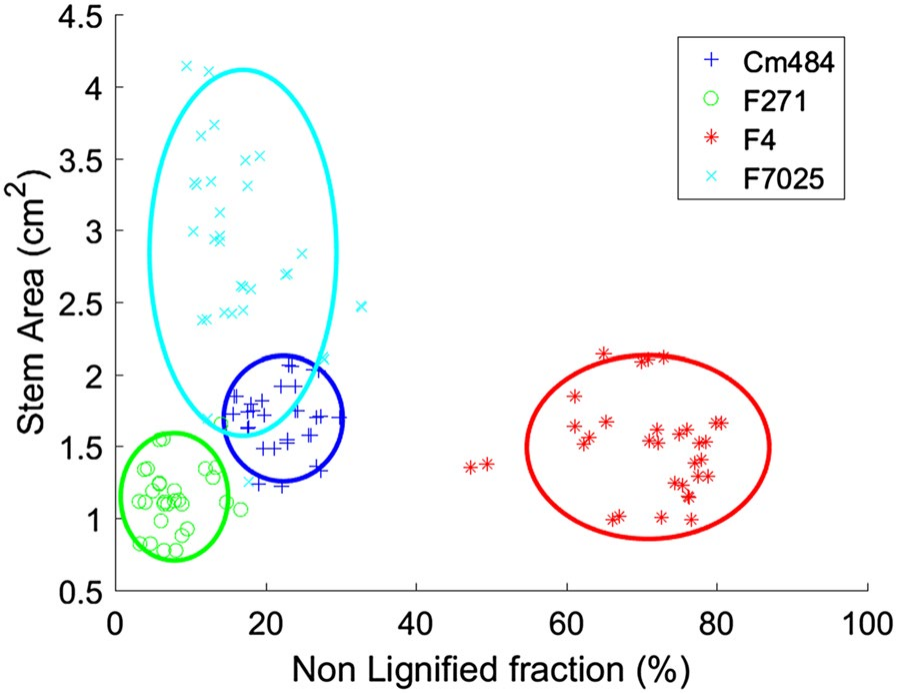
Scatter plots of the whole slide microscopy images according to the stem area and the fraction of non-lignified tissue region, grouped by genotype

The discriminative power of morphometry parameters obtained from quantitative histology is summarized on Fig. 4. The F4 genotype is characterised by a large fraction of non-lignified tissue. The size of the stem section helps to discriminate the three other genotypes.

### Comparison of water treatments

**Fig. 5:**
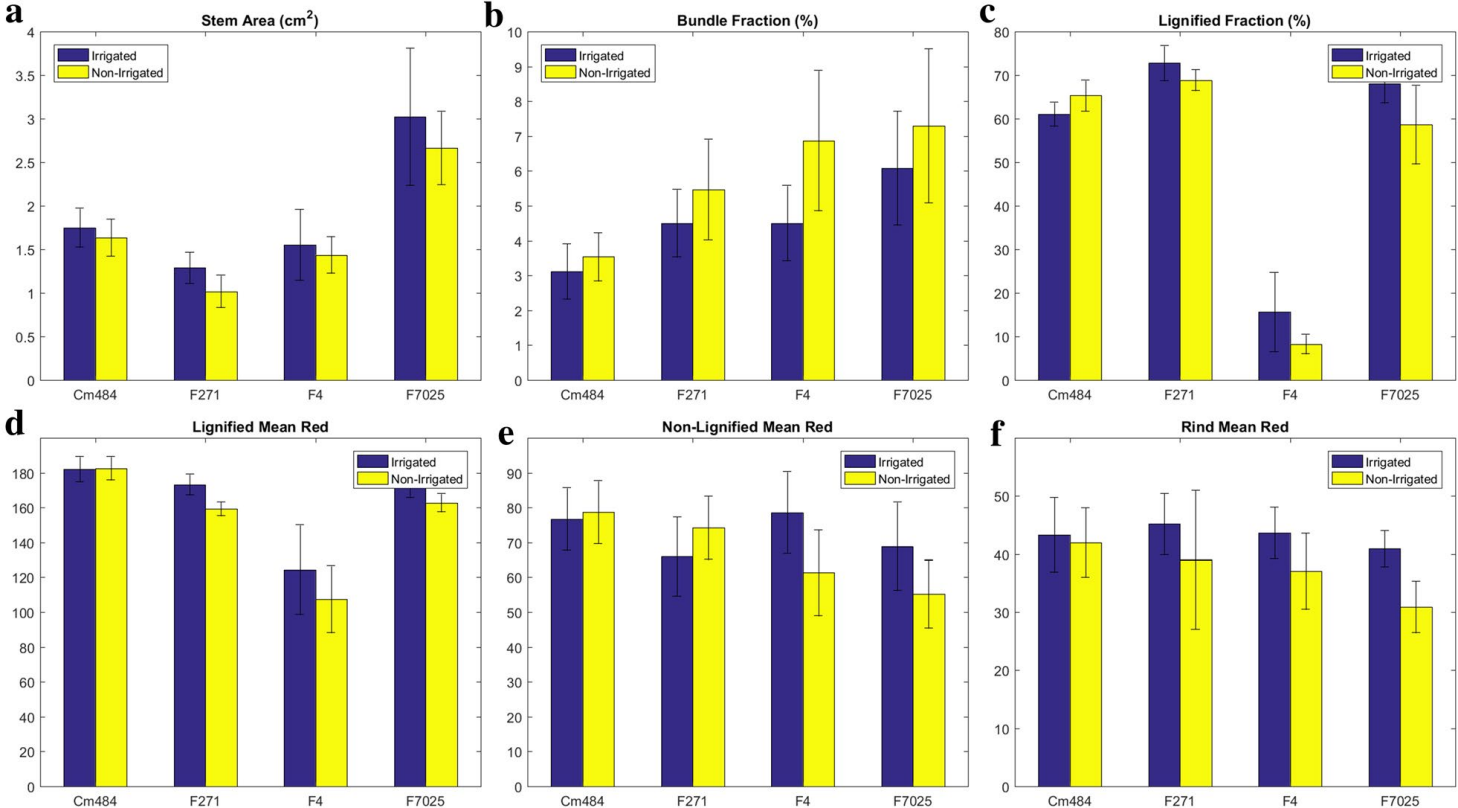
Variations of several descriptive parameters depending on both the genotype and the water treatment. **a** stem area, in cm^2^, **b** bundle fraction, **c** lignified fraction, **d** mean red value in lignified fraction, **e** mean red value in non-lignified fraction, **f** mean red value in the rind tissue region

The effect of water treatment can be observed on several parameters (Table 1 and Additional file 1). Some of them are represented in Fig. 5.

For all genotypes, the stem area of the section is smaller in the case of water deficit (*p* value of the water treatment effect ≈ 0.005) (Fig. 5a). The area fraction occupied by vascular bundles increases for all genotypes (*p* value of the water treatment effect ≈ 0.009) (Fig. 5b). Since the number of vascular bundles remains the same, it seems that water deficit favours the production of larger vascular bundles.

Several (genotype × water treatment) interactions are significant, showing a specific response of the genotypes to the water treatment (Table 1). The proportion of lignified tissue regions changes with water treatment (*p* value of the water treatment effect ≈ 0.024), but the change depends on the genotype (*p* value of the genotype-water treatment interaction ≈ 0.036). For F4 and F7025 genotypes, the fraction of lignified tissues decreases with water deficit (Fig. 5c). The effect is less visible for the F271 genotype and the opposite for the Cm484 genotype.

The coloration of the tissue regions changes with water deficit. The mean red value of the lignified fraction is globally smaller in the case of water deficit (*p* value of water treatment effect ≈ 0.003). Specific response of the genotypes can be observed (*p* value of water treatment effect ≈ 0.008), revealing that the diminution occurs for all genotypes except for Cm484 (Fig. 5d). The change in coloration with water deficit of the non-lignified tissue fraction depends on the genotype (*p* value of the interaction ≈ 0.013). The mean red value of the non-lignified tissues is smaller for F4 and F7025 genotypes, larger for the F271 genotype, and does not change for the Cm484 genotype (Fig. 5e). For all genotypes except Cm484, the mean red value of the rind tissues is smaller in the case of water deficit (*p* value of water treatment effect ≈ 0.001) (Fig. 5f).

For all genotypes, the water deficit is related to a diminution of the mean value of each colour component of the bundle fraction that can be observed as a darkening of the vascular bundles. Such darkening may be interpreted either as an increase in lignification of the cell walls or as a densification of the cell walls in the tissue region, making the coloration more difficult to quantify.

## Summary

**Fig. 6:**
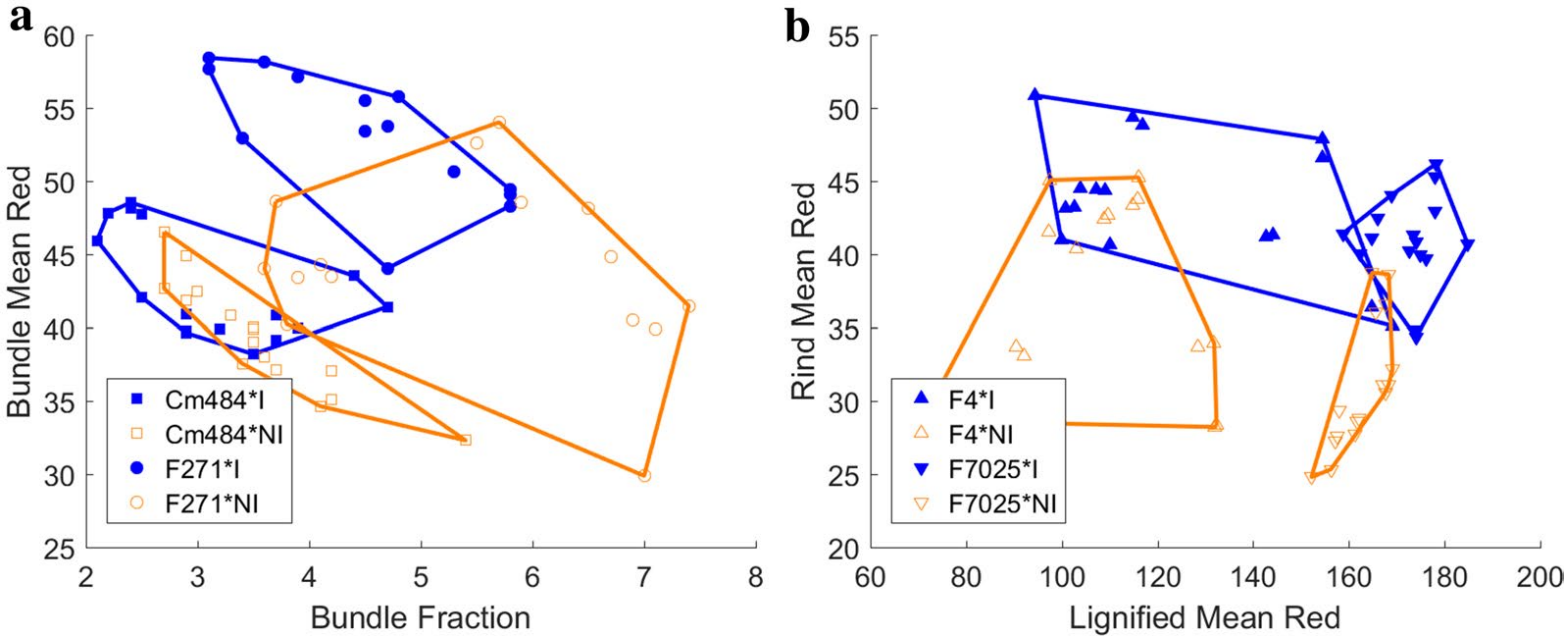
Scatter plots of the stem sections depending on specific histological parameters. **a** Effect of the water treatment on F271 and Cm484 genotypes. **b** Effect of the water treatment on F4 and F7025 genotypes

Both genotypes and water treatments can be discriminated based on parameters obtained from quantitative histology. For all genotypes, water deficit results in smaller sections and larger bundles. The bundle fraction and the mean red colour in the bundle fraction represents the differences in histology occurring in the bundle fraction for the F271 and the Cm484 genotypes (Fig. 6a). The mean red values in the lignified fraction and in the rind fraction are better suited for the representation of histological changes due to water treatments for genotypes F4 and F7025 (Fig. 6b).

## Conclusions

We have presented a complete image processing workflow for the automated analysis of images of FASGA-stained sections of maize internodes. The FASGA staining enhances the contrast of the tissues observed within the sections, and makes it possible to discriminate between lignified and non-lignified tissues. The automated segmentation procedure can identify the different regions within the section, and determine whether they are lignified or non-lignified. The quantitative characterisation results in morphometric parameters that describe the size, the proportion and the colorimetric information of each tissue.

The set of parameters can successfully discriminate contrasted genotypes, validating the proposed approach. The key parameters for discriminating genotypes are the size of the section, the relative area fractions of the tissues, the number of bundles and the colour of the different tissue fractions. Moreover, contrasted effects of the water treatment can be observed on some genotypes. Water stress seems to increase the bundle fraction in some genotypes and to decrease the lignification of the rind fraction in other genotypes. These first results are promising for studying the effects of environmental factors on the variations in chemical composition and histology of large collections of genotypes [43]. In particular, it would be of interest to verify if the effects observed on the four genotypes are observed in a wider genetic population.

The obtained results validate the use of whole slide scanners for quantitative histology of stained plant tissues. Since the images were not processed with the maximum resolution, it is expected that more precise tissue segmentation could be obtained. In particular, it may be possible to quantify the cell morphology or the cell wall thickness. The quantification of the heterogeneity of cellular morphology within the section could provide new insights into previous results [16, 31, 43, 44]. However, the large size of images obtained at full resolution (typically, several gigabytes) also complicates the development of high-throughput image analysis algorithms.

The quantitative features obtained from histological staining can also be related to the chemical composition of the tissues, such as the lignin content [31]. Acquisition devices such as confocal microscopy or microspectroscopy can provide more detailed information about the chemical content of plant tissues [13–15]. The field of view is however limited to several dozen cells. Future work may therefore focus on the fusion of information obtained from different modalities in order to calibrate high-throughput methods with acquisition methods that are more informative, but that may require more time to analyse large quantities of samples.

## Additional file

**Additional file 1.** Sample image showing the result of segmentation of tissue regions superimposed on the original image. Some vascular bundles that could not be discriminated from the rind are manually highlighted in magenta.
