# Supplementary figures and images for: Histological quantification of maize stem sections from FASGA-stained images
Source: Plant Methods. 2017 Nov 1;13:84. doi: 10.1186/s13007-017-0225-z (PMC5664815; doi:10.1186/s13007-017-0225-z)

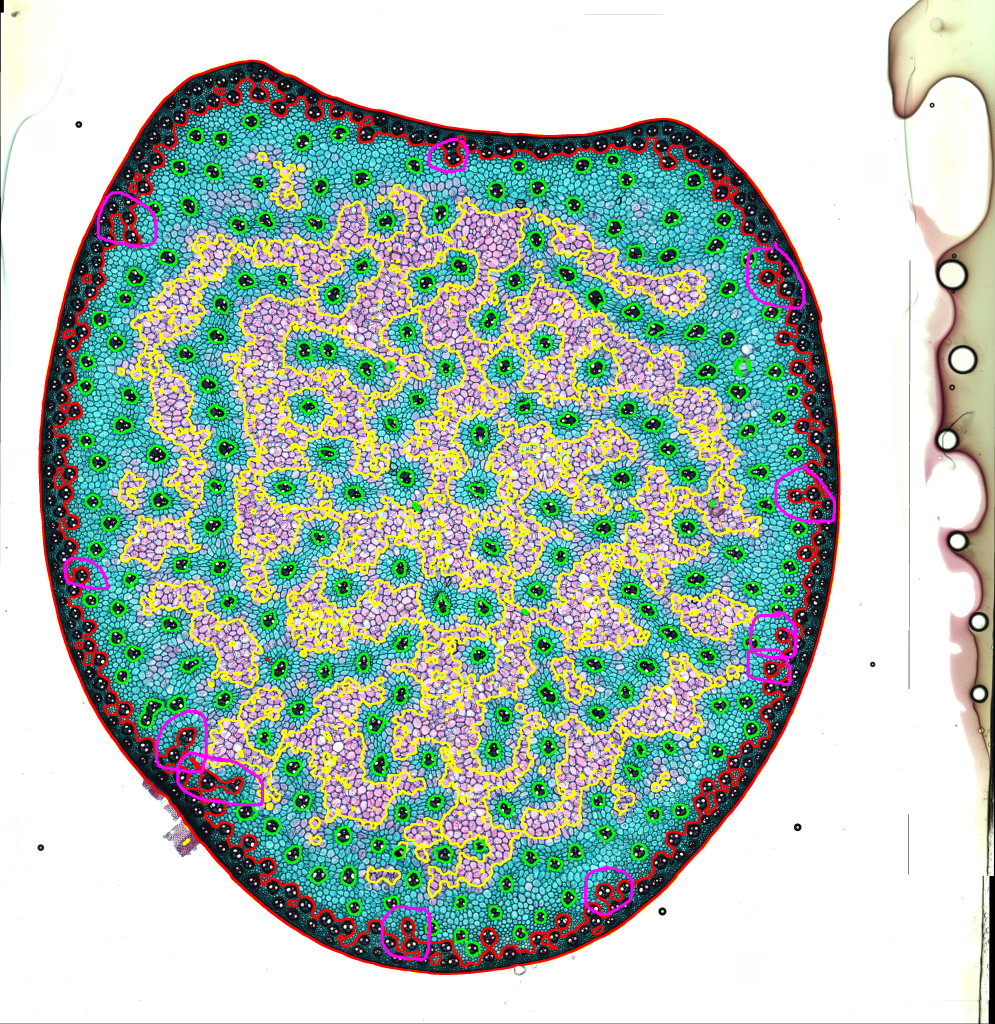

Supplement: Supplementary file 1 — Additional file 1. Sample image showing the result of segmentation of tissue regions superimposed on the original image. Some vascular bundles that could not be discriminated from the rind are manually highlighted in magenta. [file 13007_2017_225_MOESM1_ESM.tif]
